# Medical Journal Advertisements

**Published:** 1898-10

**Authors:** 


### MEDICAL JOURNAL ADVERTISEMENTS.

THE *Tri-State Medical Journal and Practitioner*, in an editorial in its August number, has this to say:

"We are grieved to notice that in the past six months the advertisement of Ayer's Cherry Pectoral has appeared in several journals which are supposed to be conducted for the regular medical profession. Among those which have lapsed from the path of rectitude we notice the following: *Medical Mirror, Gaillard's Medical Journal, Iowa Medical Journal, Charlotte Medical Journal, Cincinnati Lancet-Clinic, Kansas City Medical Index.*"

What the future of medical journalism will be is a question which only time can tell. At the present outlook the tendency of a large majority of the journals is to be run in the interest of the advertisers, and even these latter are not selected. So-called journals are even published which contain nothing but abstracts and a rehash of other medical journals, their only purpose being either to get new books from publishers, to see their names in print, or to make a dollar or two by being the medium for the advertisement of some secret nostrums. Medical journals have ceased to be published for the profession, but they are "cut-throat" advertising mediums for whatever they can get. The policy of the ATLANTA MEDICAL AND SURGICAL JOURNAL has always been the same—*i. e.*, its pages are for the ethical professional men, and its rates for legitimate advertisements are the same to all. We make no reductions. Only in the last month have we refused two advertisements because the advertisers wanted to dictate their own terms, and yet you can hardly pick up a medical journal wherein you will not find these very advertisements, and it is proof positive that the pages of those journals are open to the advertisers on the basis of "What will you give us?" There is no ethics in medical journalism. There are a few journals which we are always ready to recognize as standing solely for the good of the profession, but the great bulk of the journals which are published never for a moment have the medical profession at heart. Journals which make their living from the support furnished them through their advertising pages don't need the support of the profession, but simply send complimentary copies all over the country and then represent to the advertising agents that they have the largest circulation of any journal published. Nowhere in the world does this state of affairs exist except in the United States. Whenever you pick up a foreign medical journal you can rely upon it that its pages contain some good reading-matter. Yet we see no remedy, the only solution being that as suggested by the editorial published in our last issue from the *American Obstetrical and Gynecological Journal.*

